# Epidemiology of childhood tuberculosis and factors associated with unsuccessful treatment outcomes in Tigray, Ethiopia: a ten-year retrospective cross sectional study

**DOI:** 10.1186/s12889-019-7732-y

**Published:** 2019-10-24

**Authors:** Gebremeskel Mirutse, Mingwang Fang, Alemayehu Bayray Kahsay, Xiao Ma

**Affiliations:** 1 School of Public Health, College of Health Science, Mekelle University, Mekelle, Tigray Ethiopia; 2 Department of Health-Related Social and Behavioral Science, West China School of Public Health, Sichuan University, Chengdu, 610041 China

**Keywords:** Tuberculosis, Treatment outcome, Childhood, Tigray, Ethiopia

## Abstract

**Background:**

Childhood TB is an indicator of a recent transmission of the disease in a community and it is estimated to constitute 15–20% of all TB cases in many of developing countries. However, only few studies which dominated by industrial countries were engaged to assess the situation. Therefore, this study was aimed to see epidemiology of childhood TB and factors associated with poor treatment outcome in developing country.

**Method:**

Using retrospective cross-sectional study design; Socio-demographic and clinical data of children aged less than 15 years old, treated for all forms of TB in the past 10 years (2007–2016) was collected from randomly selected eight public hospitals of Tigray. Then, Univariate logistic regression and adjusted multivariate logistic regressions was done to identify variables which had association with unsuccessful treatment outcomes at *P*-value less than 0.05.

**Result:**

In the past 10 years, a total of 13,345 Tuberculosis cases were observed. Of these, 1086 (8.1%) cases were children aged less than 15 years old. Sixty seven (6.2%) cases were smear positive. Among those that tested for HIV, 69 (8.3%) cases were TB/HIV co-infected. Of those with treatment outcome record 746 (88.7%) were successfully treated. Factors like being female (AOR, 1.79; 95% CI, 1.07–3.00), Age 0–5 years (AOR, 3.35; 95% CI, 2.11–5.33), Unknown HIV status (AOR, 2.44; 95% CI, 1.51–3.95) and pulmonary positive case (AOR, 2.56; 95% CI, 1.13–5.77), were more likely to have unsuccessful treatment outcome than their counterparts.

**Conclusion:**

In Tigray 8.1% all TB cases were children age less than 15 years old. Childhood TB treatment outcome varied with sex, age and HIV status.

## Background

Childhood tuberculosis adds nearly 15–20% of all TB cases worldwide [1–3]. In the year 2015, one million children were ill; 200,000 died and more were left severely disabled [4]. The diagnostic difficulties of childhood TB, the child-unfriendly drug formulations together with the inclination of tuberculosis control strategy toward adult [5] ended children younger than 15 years old to remain under-diagnosed and with poor treatment outcome respectively [2, 6].

TB can imitate sign and symptoms of many common childhood diseases, including pneumonia, malnutrition, and HIV infection which pose diagnostic difficulties. However, the main obstacle of exact diagnosis of active TB is the paucibacillary nature of the disease in children [7].

Children living in developing country had devastating impact of tuberculosis [3, 4]. Because, the region had 28% of the world’s TB cases and 98% of deaths from TB worldwide [8, 9].

There is difficulty in defining global epidemiology of childhood tuberculosis since there is ongoing transmissions of TB [10] and the difficulty was more in African countries [11]. Ethiopia is one of the Africa counties [5] and according to The WHO estimate, TB case-detection rate was 60% which shows 40% case detection gap means every year 80,000 TB cases remained un-diagnosed [12]. Though, the share of children age less than 15 years old was expected to be big [13].

Currently the prevalence of tuberculosis in Ethiopia for general population was 192/100,000 [13, 14]. Also, data collected from public hospitals in Ethiopia revealed that TB as the leading cause of morbidity and the second cause of death after malaria [5].

Magnitude of childhood tuberculosis was not known in Africa including Ethiopia. And its reason was the lack of consistent report. But, few report indicate in the year 2012 total of 19,500(14%) children infected with tuberculosis was reported by NGO called MSH [15, 16], in Malawi 12% of all the cases and in South Africa children contributed to 15% of the burden [2, 17].

The implementation of Stop TB Strategy, founded on the Directly Observed Treatment (DOTS) saved 250,000 children [2]. However, some believe like childhood TB is not important for TB control and the difficulty of diagnosing childhood TB made the prevention progress less promising [5, 11]. Hence, to tackle this problem, the World Health Organization developed a road map aiming to achieve zero deaths due to childhood TB by 2025 [6] and made a call for more studies to be done on childhood TB since, they were scant [5, 11].

Proclaiming the above reasons, investigating age and sex-aggregate data maintained by TB programs will be helpful to specify the magnitude, profile, treatment success and its factors affecting treatment outcome of childhood.

## Methods

### Study locations and DOTs service

This study was conducted in the Tigray regional state which is located in the northern part of Ethiopia [18] with population size of 5.1 million. In this region the tuberculosis diagnosis and treatment protocol, and DOTs services were not a centralized type. Hence, all hospitals and health centers are allowed to diagnosis TB cases as well provide DOTs service in their site [19]. In this region there are a total of 16 functional public hospitals and 204 health centers [20].

### Design of the study and data collection

Using retrospective cross-sectional study design we reviewed all children treated for all form of TB from the years 2007 to 2016. In this region there were 16 public hospitals which provide DOTs service. Then, among these all, eight hospitals were randomly selected by lottery. Finally, trained data collector and supervisors were sent to the area to collect Socio demographic and clinical information (Type of TB pulmonary positive or negative and extra pulmonary; HIV status Treatment outcome (cured, complete, default, failed, died) of children treated in the past 10 years from TB patient’s registry.

### Analysis

Data was entered in to SPSS Version 21 then, descriptive analysis such as frequency, mean and standard deviation were computed. Furthermore, the dependent variable treatment outcome was categorized in to successfully treated and unsuccessfully treated. Then, Univariate logistic regression was done to identify variables which had association with unsuccessful treatment outcomes at *P*-value less than 0.05. Finally, variables significant at *P*-value less than 0.05 values in the unadjusted Univariate analysis were selected and analyzed on the adjusted multivariate logistic regressions.

### Operational defections

The following clinical and treatment outcome definitions were taken from the standard operational definitions of Ethiopia National Tuberculosis and Leprosy Control Program guideline (NLCP) [19].

#### Childhood tuberculosis

A person whose age was 0–14 years old and was diagnosed as TB cases as well treated for TB.

#### Smear positive tuberculosis

A patient with at least two initial sputum smear examinations positive for AFB by direct microscopy, Or A patient with one initial smear examination positive for AFB by direct microscopy and culture positive.

#### Smear negative tuberculosis

A patient having symptoms suggestive of TB with at least 3 initial smear examinations negative for AFB by direct microscopy, and no response to a course of broad-spectrum antibiotics.

#### Extra-pulmonary TB (EPTB)

TB in organs other than the lungs, proven by one culture-positive specimen from an extra-pulmonary site or histo-pathological evidence from a biopsy,

#### Cured

A initially smear-positive patient who is sputum smear-negative at, or one ‘month’ prior to, the completion of treatment and on at least one previous occasion (usually at the end of the 2nd or 5th month).

#### Completed treatment

A patient who completed treatment but for whom smear results are not available at 7th month or 1 month prior to the completion of treatment.

#### Treatment failure

A patient who remains or becomes again smear-positive at the end of 5 “month” or later during treatment.

#### Lost to follow-up

A TB patient who did not linked to start treatment or interrupted his treatment for two consecutive months or more.

#### Not evaluated

A TB patient for whom no treatment outcome is assigned. This includes cases ‘transferred out’ to another treatment unit as well as cases for whom the treatment outcome is unknown to the reporting unit.

#### Successful treatment outcome

The sum of TB patients who declared “cured” and those who have “completed” treatment.

#### Unsuccessful treatment outcome

Patient who died from TB during the course of treatment, interrupted treatment for two consecutive months or more after registration, patient remaining smear positive at 5 months despite correct intake of medication.

#### Died

A TB patient, who died from any cause during the treatment period.

## Results

### Over all profile of study participant

In the past 10 years (September 2007 to August 2016,) a total of 13,435 TB cases treated in the region among these 1086(8.1%) cases were children age less than 15 years old. The mean age of children was 8 years with (SD ± 4.09), majorities were new case 1082(99.7%), Females 701 (64.5%), and with a form of extra pulmonary tuberculosis 678(62.4%). Moreover, 843 TB cases were tested for HIV and 69 (8.3%) of the case were TB/ HIV co infected (See in Table 1).

**Table 1:** General Profile of Childhood Tuberculosis in the past 10 years Sep 2007-Aug 2016 in Tigray, Ethiopia *N* = 1086

Sociodemographic and clinical factors	Number	Percent
Age		
0-5 years	330	30
6–14 years	760	70
Sex		
Female	710	64.5
Male	376	35.5
Residence		
Urban	457	42.6
Rural	629	58
TB type		
P. Positive	67	6.2
P. Negative	341	31.4
Extra Pulmonary	678	62.4
HIV Status		
Negative	774	71.3
Positive	69	6.3
Unknown	243	22.4

### Cases included in the analysis and treatment outcomes

**Fig. 1:**
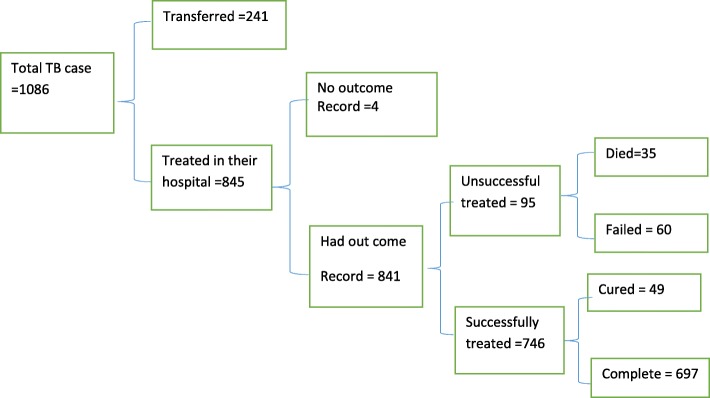
Treatment outcome of childhood Tuberculosis in Tigray, Ethiopia September 2007–August 2016 *N* = 1086

Within the study period, a total of 1086 case age less than 15 years were observed in eight hospitals. Of those, in their treatment outcome record we observed, 241(22.2%) cases as transfer, 4 (0.04%) cases with unknown treatment outcome and a total of 841(77.4%) cases had known treatment outcome record. Of these 95(11.3%) cases were unsuccessfully treated and 35 (4.2%) cases were died. (See in Fig. 1).

### The pattern of tuberculosis treatment success and death

**Fig. 2:**
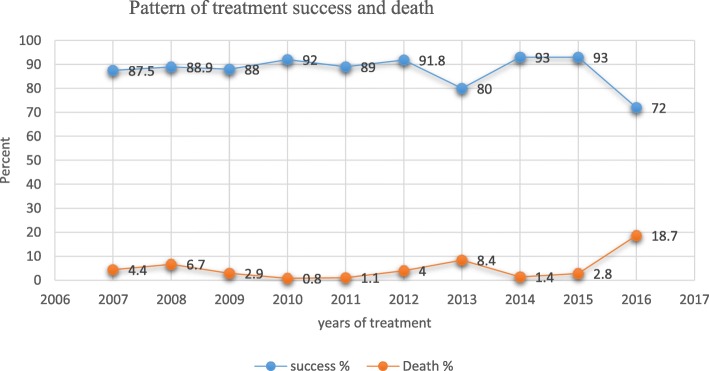
TB cases successfully treated and death in the Past 10 years September 2007–August 2016 Tigray, Ethiopia. *N* = 841

In the past 10 years among 1086 observed cases, a total 841 TB cases had treatment outcome records and of those with treatment outcome 746(88.7%) cases were successfully treated. In these period of study, the lowest treatment successes were seen in the year 2016 and 2013 which is 72 and 80% respectively. Similarly, the highest deaths were seen in the year 2016, 2013 and 2008. In this study period, a total of 35 (4.2%) TB cases were died and majorities 26 (74%) of them were females. (See in Fig. 2).

### Factors associated with unsuccessful treatment outcomes in children

Unadjusted bivariate and adjusted multivariate logistic regressions were done to identify variables which have association with unsuccessful treatment outcome. Thus, unadjusted bivariate analyses indicated that, those TB cases, like females, age between 0 and 5 year’s old, unknown HIV status, pulmonary negative cases and cases living in rural area were more likely to have unsuccessful treatment outcome.

Holding other factors constant (sex, age, residence, HIV/AIDS type of TB pulmonary v. extra pulmonary). TB cases who are females were 1.79 times more likely to have unsuccessful treatment outcome (95% CI, 1.07–3.00) compared with male. Children in the age category of 0–5 were 3.35 times more likely to have unsuccessful treatment outcome (95% CI, 2.11–5.33) compared with age category of 6–14. Similarly, children with unknown HIV status 2.44 times more likely to have unsuccessful treatment outcome (95% CI, 1.51–3.95) comparing with HIV negative and positive cases. Finally, pulmonary positive cases were 2.56 times (95% CI, 1.13–5.77), more likely to have unsuccessful treatment outcome compared with extra pulmonary cases. (See Table. 2).

**Table 2:** MLR: Socio demographic and clinical factors associated with unsuccessful treatment outcome among childhood tuberculosis September 2007–august 2016 Tigray, Ethiopia. *N* = 841

Socio and clinical factors	Number of cases		Unadjusted		Adjusted by age, sex, HIV and TB type	
	Total	Unsuccessful (%)	OR^a^	95% CI	OR^a^	95% CI
Sex						
Female	560	72 (12.8)	1.65	1.01–2.71^a^	1.79	1.07–3.00^b^
Male	281	23 (8.1)	1		1	
Age category						
0–5	268	55 (20)	3.44	2.22–5.33^a^	3.35	2.11–5.33^b^
6–14	573	40 (6.9)	1		1	
Residency						
Rural	467	63 (13.6)	0.60	0.38–0.94^a^	0.65	0.40–1.03
Urban	374	32 (8.5)	1		1	
TB type						
P-Positive	60	9 (15)	1.88	0.87–4.07	2.56	1.13–5.77^b^
P-Negative	256	41 (16)	2.03	1,29–.3.19^a^	1.84	1.14–2.97^b^
E. pulmonary	525	45 (8.5)	1		1	
HIV status						
Unknown	188	36 (19)	2.37	1.50–3.76^a^	2.439	1.507–3.95^b^
Positive	57	5 (8.7)	0.96	0.37–2.52	0.76	0.28–2.05
Negative	596	54 (9)	1		1	

*COR* Crude odds ratio^a^ significant, *AOR* Adjusted odds ratio^b^ significant

## Discussion

An accurate estimate of the global burden of tuberculosis in children is difficult mainly because of the challenges in case ascertainment, diagnosis, and weak surveillance systems in many countries with a high burden of tuberculosis [2] Hence, to define the global epidemiology of childhood TB and obtain due attention, the WHO has call for more studies [5]. Thus, in response to the call, this retrospective study was done in Tigray regional state which located in the northern part of Ethiopia.

In Tigray, in the past 10 years a total of 13,435 all age of Tuberculosis cases were observed and of theses 1086 (8.1%) cases were children age less than 15 years. This finding is lower than the report of MSH (14%) [4], a review study done by Getahun et al., retrospective study report from southern Ethiopia (13%) and WHO 2015 report (16%_20%) [9, 14, 21]. This indicates that in Tigray childhood TB was under diagnosed for the past 10 years. The reason could be misconceptions in the community like that infants and young children are a particular low risk group for severe disease because materials that informs about the specific challenges of tuberculosis in children which support community engagement are little. As well contact screening by health workers could be low in the region [22] and the uncomfortable diagnostic procedure of TB for children.

Gender difference in TB infection was not clearly understood. However, in this study majority of Tuberculosis cases were female 710 (65.4%) and this finding was similar with a prospective study done in India 61.7% of cases were Female [23] and a retrospective study done in Addis Ababa 55% [5]^.^ This indicates that in the period of childhood females are more affected by Tuberculosis than male. Yet, the reason was not clear why girls more affected than boys in the period of childhood.

The magnitude of all from of Tuberculosis in the age group of 0–5 years old was 330 (30.4%) which was higher than a study done in Addis Ababa 11.4% and Congo 24% [12]. But, lower than the study done in Sudan 46.7% in the age of 0–4 years [24]. This dis similarity could be children in Tigray and Sudan had uncontrolled frequent contact with adult infectious TB patents.

Worldwide, extra-pulmonary TB occurs in 10 to 42% of patients. But, in TB-endemic developing countries since childhood TB diagnosis depends on clinical characteristics and subjective interpretation of chest X-ray the size of the extra-pulmonary cases were big [25]. Again some clinician did not follow the childhood tuberculosis diagnosis organogram. Therefore, in this study, the size of the extra-pulmonary case were 678 (62.4%) which is consistent with a study finding of Addis Ababa 52.8% [5, 24].

In TB epidemic countries TB/HIV co infections was common. In this study, among 843 TB case which tested for HIV 69 (8.3%) cases were found as HIV positive and it was equivalent with finding of Sudan 6.2% which studied by WHO. But, lower than Congo 19.4%(12)and Addis Ababa 16.7% [5]. The reason may be there was decline in the incidence of HIV from time to time in the region. Other reason could be Addis Ababa and Cong are the capital city of the country which had tertiary hospitals in which more complicated TB case and HIV/ co infected cases were referred for treatment.

Children with TB, usually have an excellent clinical outcome if diagnosed in a timely fashion and treated appropriately. In our study 746(88.7%) case were successfully treated, this finding was consistent with retrospective study report from Addis Ababa 85.5% percent [5] and The WHO estimate [26]. Hence, the reason to had equivalent level of successful treatment outcome may be there was timely diagnosis and better drug adherence in these regions.

In this study among total cases who have treatment outcome records 35 (4.2%) children were died and it is higher than retrospective study done in Addis Ababa indicates 2% (5)and study report from Sidama 3% [21]. But, similar with the study done in Sudan 4.3% [24]. This high rate of death could be the region had high magnitude of child under nutrition which is 33% of children were underweight and 11.6% were wasted [27]. Hence, this condition had high probability to complicate the outcome of treatment.

In the adjusted multivariate analysis TB cases who are females were 1.79 times more likely to have unsuccessful treatment outcome (95% CI, 1.07–3.00) compared with male. But, study done in Addis Ababa shows no significant difference [5]. This high rate of unsuccessful treatment in our study may be the number of HIV positive and unknown HIV status female cases are double of those males this may have an effect on the outcome of treatment.

Children in the age category of 0-5 years old were 3.35 times more likely to have unsuccessful treatment outcome (95% CI, 2.11–5.33) compared with age category of 6–14. This could be due delayed health seeking behavior of the community which leads to the late diagnosis and late initiation of anti TB treatment.

Similarly, children with unknown HIV status were 2.44 times more likely to have unsuccessful treatment outcome (95% CI, 1.51–3.95) compared with HIV negative and positive cases. This is similar with study done in Addis Ababa unknown HIV status (AOR = 0.94 (0.57–1.68) had significantly lower treatment success rate [5]. The TB/ HIV prevention and treatment guideline indicates that any TB case should be tested for HIV and linked to Pre-ART treatment. But, some families reject the HIV testing offer and delay the child to take correct treatment protocol as well some health facility forgot to offer the test and delay in linking to ART program.

Finally, pulmonary positive cases were 2.56 times (95% CI, 1.13–5.77), more likely to have unsuccessful treatment outcome compared with extra pulmonary cases. In the past two decades there was a widespread of drug resistance TB in the region. But, drug resistance checkup was initiated lately in the region. So, these miss children with MDR cases which lead to poor outcome of pulmonary positive TB.

This study had limitations one many TB cases were excluded from the analysis because of their transfer to other unknown facilities. Another limitation was the absence of micro-biologic confirmation in most diagnosed patients.

## Conclusions

In Tigray among all TB cases 8.1% are children age less than 15 years old. Childhood TB treatment outcome varied with sex, age and HIV status in Tigray regional state. Hence, comparing with global estimated report there was under diagnosis in the region Therefore, intensified effort should be consider mitigating under diagnosis in the region.

## Data Availability

The data that support the findings of this study are available from the corresponding author, [xiao Ma], upon reasonable request.

## Abbreviations

AOR: Adjusted Odds Ratio MSH: management Science for health
MDR TB: Multi Drug Resistance Tuberculosis
TB: Tuberculosis
WHO: World Health Organization
